# Religious headwear and alopecia: considerations for dermatologists

**DOI:** 10.1097/JW9.0000000000000107

**Published:** 2023-09-06

**Authors:** Lina Alhanshali, Michael G. Buontempo, Fatima Bawany, Prince Adotama, Jerry Shapiro, Kristen Lo Sicco

**Affiliations:** a Department of Dermatology, SUNY Downstate College of Medicine, Brooklyn, New York; b Department of Dermatology, Hackensack Meridian School of Medicine, Nutley, New Jersey; c The Ronald O. Perelman Department of Dermatology, NYU Grossman School of Medicine, New York City, New York

**Keywords:** alopecia, headwear, hijab, kippah, peyot, religion, turban

**What is known about this subject in regard to women and their families?**

- Religious head coverings are worn by many people throughout out the world, mainly women.
- Religious headwear may be associated with alopecia although there is limited research on this topic.

**What is new from this article as messages for women and their families?**

- Alopecia secondary to religious head coverings is due to the ways in which the headwear is worn and hairstyle practices underneath.
- There are ways to mitigate the risk of alopecia in people who wear religious head coverings without compromising religious beliefs.

An estimated 84% of the global population identifies with a religious group.^1^ Wearing religious headwear has been associated with alopecia, most notably traction alopecia (Fig. 1). Traction alopecia can result from how the headwear is secured and styled, or other practices that may lead to tension on the hair and scalp. Individuals with alopecia who observe religious head coverings may struggle to conform to practices regarding headwear styling. As this topic is scarcely reported in the scientific literature, the authors relied on their personal experiences and expertise in treating hair and scalp disorders to provide recommendations for mitigating alopecia risk.

**Fig. 1.**
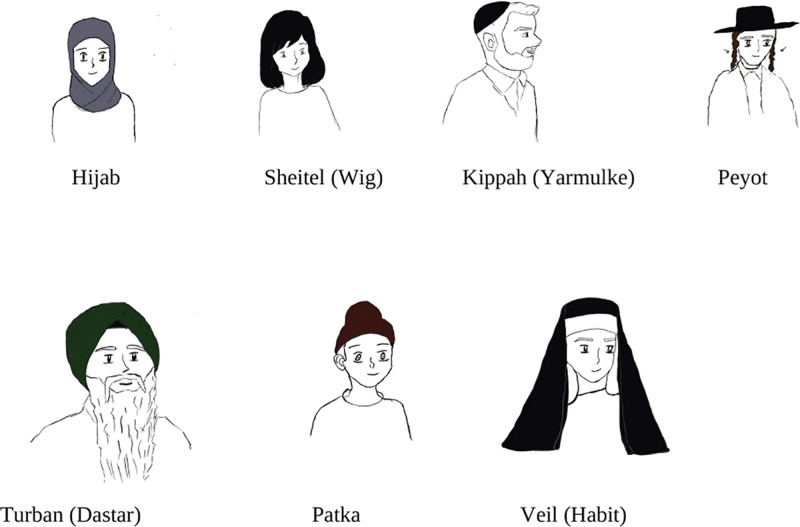
Various religious headwear practices.

Although no published clinical study directly implicates religious headwear in alopecia presentation, there is a strong discourse on headwear and hair loss. For example, Shareef et al.^2^ analyzed self-reported hijab-related alopecia content on YouTube and found 27 videos with a total of 17,158,078 views. Traction is a likely contributor to hijab-related alopecia, owing to the consistent and tight wrapping of the hijab around the head and hair, which can lead to continuous pull on the hair roots. This is a particularly important consideration for certain subgroups, such as women with afro-textured hair who have fewer elastic fibers attaching hair follicles to the dermis compared to Caucasians.^3^

In the Jewish tradition, wigs, known as sheitels, worn by Orthodox women may cause traction alopecia if they create tension on the scalp, particularly from wig attachment techniques. Wig placement often requires natural hair to be styled in a sleek, compact manner, promoting tight hair styling techniques such as buns and braids that can lead to alopecia. A case series on dermatologic considerations in ultra-Orthodox Jews reported a woman who presented with an alopecic patch in the frontal scalp where the sheitel clip was consistently placed.^4^ Hair regrew in the region after the patient transitioned to a clipless wig. Similarly, a study of 37 men with localized alopecic patches in the region of the pins used to secure their kippah showed that 58.8% of the patients who changed to a different type of pin fastener exhibited hair regrowth.^5^

Traction alopecia related to the turban or dastar, worn by Sikh men, has been reported in the scientific literature and is referred to as turban alopecia.^1^ This condition results from the tension caused by hairstyling and/or headwear styling. Sikh men generally style the hair in a bun or knot that is wrapped tightly with a cloth or scarf around the head. Tension can result from the bun itself, or from the turban being worn too tightly.

The Rastafarian tradition encourages the growth of dreadlocks, which are considered a symbol of the Lion of Judah and a mark of African identity. The risk of traction alopecia can be influenced by the weight and length of dreadlocks as well as the tightness with which they are tied or wrapped under a headwrap.

Recommendations for preventing and treating religious headwear-associated alopecia are outlined in Table 1. Alopecia related to religious headwear is a significant concern affecting a large population in the United States and globally. Dermatologists should be aware of risks and cultural nuances when treating patients from diverse religious backgrounds. By incorporating culturally competent care, dermatologists can effectively address and prevent headwear-associated alopecia, fostering patient trust and well-being.

**Table 1:** Actions and tips for mitigating headwear-associated alopecia and cultural sensitivity recommendations

Religious group	Headwear	Mitigating alopecia actions and tips	Cultural sensitivity tips for the patient–clinician interaction
Islam	Hijab	Increase awareness and knowledge, including that traction alopecia is potentially irreversible when chronic Recommend hijab fabrics that are less likely to slip (such as jersey or cotton) Recommend under caps that can be tied to the back or allow for size adjustment as opposed to one-size, tube-like under caps If hair under hijab is styled in a knot or ponytail advise to vary the location in which it is styled	Muslims may not make physical contact with the opposite gender including shaking hands Muslim women may prefer to see a female physician. Male physicians can inquire if a female patient would like a chaperone present Recognize that although Muslim women cover their hair in public, hair is still important to their identity, and alopecia may significantly impact their quality of life
Judaism	Sheitel	Increase knowledge and awareness of alopecia Mindfulness of wig attachment techniques If wearing wigs with clips, advise to alternate areas of the scalp Provide information on the types of wigs available	Orthodox Jews may not make physical contact with the opposite gender such as shaking hands Male physicians can inquire if a female patient would like a chaperone present Jewish patients may not be able to use electronic therapies such as lasers during Sabbath observance (Friday night at sundown until Saturday evening at sundown)
	Kippah	If using pins to secure kippah, advise to alter the location of the pins on the scalp	Jewish men who experience hair thinning or alopecia at or near crown may have difficulty keeping kippah in place. Advise to use double sided tape^a^, adhesive strips^a^, large-sized kippahs, or knit kippahs
	Peyot		It is customary for peyot (long sideburn hairs) to be of the same length. In a person experiencing alopecia, one may become short It is not permissible for peyot to be cut
Sikhism	Turban (Dastar) Patka	Recommend refraining from wearing turban or patka during times when it is not mandated such as during sleep Advise to leave hair out when not wearing turban or patka. If the hair is too long and bothersome, a low ponytail or braid is an alternative to counteract tension in the scalp	It is not permissible for hair to be cut
Christianity	Veil (Habit)	Advise to refrain from wearing the veil tightly Recommend leaving the hair loose in private	
Rastafarianism	Dreadlocks and headwraps	Gentle handling of hair Advise to avoid twisting hair too tightly	It is not permissible for hair to be cut

The authors relied on their personal experiences to provide this information.

a Adhesive strips on hair may cause alopecia.

## Conflicts of interest

J.S. is a consultant for Aclaris Therapeutics, Incyte, and Replicel Life Sciences. J.S. and K.L.S. have been investigators for Regen Lab and are investigators for Pfizer. K.L.S. is a consultant for Pfizer and Aquis. The other authors have no conflicts of interest to disclose.

## Author contributions

All authors contributed to the design of the study. LA conducted the literature review and wrote the manuscript. All authors contributed to critical revisions, data analysis, and interpretation. All authors approved the final version of the manuscript for submission.

## Acknowledgments

Figure 1 was created using the digital art programs Procreate and Vectornator.
